# Development of a Ready-to-Eat Fish Product Enriched with Fish Oil Entrapped in a κ-Carrageenan Egg White Fish Protein Hydrolysate Dry Powder

**DOI:** 10.3390/foods12112272

**Published:** 2023-06-05

**Authors:** María Carmen Gómez-Guillén, Selene Pérez-García, Ailén Alemán, María Elvira López-Caballero, Carmen G. Sotelo, María Pilar Montero

**Affiliations:** 1 Department of Meat and Fish Products, Institute of Food Science, Technology and Nutrition (ICTAN-CSIC), José Antonio Novais 6, 28040 Madrid, Spain; mc.gomez@csic.es (M.C.G.-G.); selene.perez.g@csic.es (S.P.-G.); ailen@ictan.csic.es (A.A.); elvira.lopez@ictan.csic.es (M.E.L.-C.); 2 Marine Research Institute (IIM-CSIC), Eduardo Cabello 6, 36208 Vigo, Spain; carmen@iim.csic.es

**Keywords:** fish valorisation, fish oil entrapment, ω-3 fatty acids, functional food, ready-to-eat, sustainable production

## Abstract

This work describes the development of a ready-to-eat (RTE) product based on an equal mixture of fish mince from three undervalued fish species with different fat contents and protein gelling capacity, which was enriched with fish oil entrapped in a κ-carrageenan egg white fish protein hydrolysate powder, obtained by either spray drying (SD) or heat drying (HD) at 80 °C (HD80). Previously, the spray-dried (SD) powder and heat-dried powders obtained at 45 °C, 60 °C and 80 °C (HD45, HD60 and HD80) were characterised in terms of water solubility, lipid oxidation (TBARS), hygroscopicity and ζ potential. All HD powders showed higher hygroscopicity and lower TBARS than the SD powder. The dry powder was incorporated into a blend composed of salt-ground batter and raw mince to improve binding and textural properties. Changes in water-holding capacity, colour, shear strength and microorganisms were monitored during the processing steps. The RTE product presented a high protein content and a noticeable amount of long-chain ω-3 fatty acids. The use of undervalued fish species together with fish oil and a protein hydrolysate from fish waste contribute to improving the sustainability of fishery resources, being conducive to obtaining a potentially functional RTE product.

## 1. Introduction

The increase in the population growth rate in recent decades that also demands more seafood products [1], the impacts of climate change on marine resource availability [2] and the need for protecting fish resources by using more sustainable practices have led to investigations of new alternatives for implementing the so-called circular economy principles in the fishery and food industries. The use of underutilised/undervalued species and the valorisation of industrial residues for their reincorporation into the production process are key factors for sustainability. Restructured seafood products represent a good example of how non-target fish species with a low commercial value can be used to design functional foods enriched with bioactive compounds from industrial waste. In this sense, grey gurnard (*Eutrigla gurnardus*) has been historically considered a discarded species; however, there has been an increase in its abundance and use interest, possibly associated with the overfishing of the target gadoid species [3]. Likewise, pouting (*Trisopterus luscus*) is another example of an underutilised fish species that has gained interest for its nutritional value and potential use as a raw material in the development of ready-to-eat (RTE) seafood products [4]. Atlantic horse mackerel (*Trachurus trachurus*), in contrast, is a popular commercial fatty fish, relatively inexpensive due to its strong flavour and dark colour. For this reason, it has been widely studied for the production of surimi and restructured products, although its high fat and protease contents generally result in very weak and oxidation-prone gels [5]. The intentional mixing of species to produce restructured products can be a strategy of great industrial interest in order to overcome the seasonality of some species and to standardise certain properties, such as texture, colour and nutritional quality [6], which could be strongly affected depending on fat content and protein-gelling capacity. A number of restructured RTE seafood products have been proposed in the recent literature, most of them based on a single fish species to which different functional ingredients are added [7,8,9,10].

The microencapsulation of fish oil into a biopolymer powder represents a widely used strategy to enrich functional foods with long-chain (LC) ω-3 fatty acids (EPA + DHA) as it overcomes strong unpleasant flavours and colours [11]. Furthermore, the use of gelling polysaccharides to entrap natural sources of ω-3 fatty acids and antioxidants was also found to be a good strategy to produce well-structured surimi gel-based matrices [12]. The relationship between the consumption of LC ω-3 PUFA and the prevention of cardiovascular disease is very well established, being preferable to increase the intake of ω-3 fatty acids through a food-based approach [13]. Microparticles based on the formation of strong covalent and non-covalent polysaccharide-protein complexes with enhanced water resistance [14,15] may be advantageous to prevent an early release of fish oil during food processing [16] and also to protect ω-3 PUFA from harsh stomach conditions [17]. A mixture of salt-soluble fish muscle protein, a fish protein hydrolysate (FPH) and κ-carrageenan was recently used for fish oil microencapsulation by spray drying or heat drying, rendering dry powders with ≈50% water solubility and ≈80% entrapment efficiency [18]. Based on these results, the present study aimed to produce alternative dry powders by replacing the salt-soluble fish muscle protein with another protein source with high water solubility and thermal gelling capacity, as is the case of egg white [19].

On the other hand, in the production of restructured fish products, polysaccharides and proteins are typically added to favour binding and texturising properties [20]. In this regard, we hypothesized that additions of κ–carrageenan, and egg white to encapsulate omega-3 rich lipids, could be good candidates with a dual purpose, i.e., as fish oil entrapment agents and product texturizers usually required to restructure fish mince and thus minimizing the required amount of additives. The inclusion of fish protein hydrolysate in the dry powder would add potential bioactive properties masking strong flavours (off-odour).

Thus, the objectives of this work were:

(i) to obtain and characterize different dry powders composed of κ-carrageenan, egg white and fish protein hydrolysate to encapsulate fish oil, by both spray drying and heat drying at different temperatures;
(ii) to study the effect of the addition of selected dry powders (spray-dried and heat-dried) on the development and properties of a ready-to-eat fish product, using a mixture of fish mince from three examples of undervalued lean, fatty and semi-fatty fish species, seeking a balanced composition in terms of fat content and protein functionality.

## 2. Materials and Methods

### 2.1. Preparation of Fish Mince

Grey gurnard (*Eutrigla gurnardus*), Atlantic horse mackerel (*Trachurus trachurus*) and whiting-pout (*Trisopterus luscus*) were caught in the North Atlantic Ocean and kept at 4 °C for approximately 24 h before being beheaded, eviscerated and washed with tap water. The minced muscles from each species (30 kg for each species), resulting from the mechanical removal of skins and bones using a deboning machine at IIM-CSIC, were frozen and sent to ICTAN-CSIC, where they were stored for about one month at −20 °C until use. The experiment was repeated three times.

### 2.2. Proximate Composition

The proximate composition was determined on the three fish mince studied separately, as well as on the finished RTE product. Moisture and ash contents were determined according to AOAC official methods 950.46 and 942.05, respectively [21]. Fat content was determined following the Bligh and Dyer method [22]. The total protein was determined with a LECO-FP 2000 nitrogen/protein analyser (LECO Corp., St. Joseph, MI, USA), using a nitrogen-to-protein conversion factor of 6.25. The total carbohydrate content was calculated by difference with respect to the total 100%, that is, by subtracting the measured protein, fat, ash, and water from the total weight. The analyses were carried out using 100 g of sample for each replicate; determinations were carried out in triplicate.

### 2.3. Gelling Properties

The gel-forming capacity of the different fish mince was determined by changes in the viscoelastic properties (elastic modulus G′ and viscous modulus G″) of the muscle protein solubilised with salt (salt-ground muscle system) as a function of heating temperature. In order to solubilise the muscle protein, the semi-thawed fish mince was homogenised with 1.5% NaCl (*w*/*w*) (Panreac, Montplet and Esteban S.A., Barcelona, Spain) for 2 min in a Stephan UM5 (Hameln, Germany) under vacuum and refrigeration. Crushed ice was added to obtain a final moisture content of 76%. Rheological tests were performed in triplicate using a Bohlin rheometer (Bohlin Instruments Ltd., model CVO, Worcestershire, UK) with a cone-plate geometry (40 mm diameter, 4° cone angle, gap = 0.15 mm). The dynamic temperature sweep was performed by heating from 10 °C to 85 °C at a scan rate of 1 °C/min, frequency of 1 Hz and target strain γ = 0.005 (within the linear viscoelastic region). The results were the mean of three determinations.

### 2.4. Entrapment of Fish Oil

Skin and bone discards from grey gurnard were ground and hydrolysed with Alcalase 2.4 L (Novozymes, Nordisk, Denmark) for the joint production of fish oil (FO) and a fish protein hydrolysate (FPH), as described in a previous work [18]. The FO was protected with 100 ppm of α-tocopherol and the FPH was freeze-dried and vacuum-packed. Both the FO and FPH were stored at −20 °C until use. The fatty acid composition of the FO, as well as the proximate and amino acid composition of the FPH, have already been reported [18].

A mixture of κ-carrageenan (C) (Trades S.A., Barcelona, Spain), egg white (EW) (Ovopack^®^, Sevilla, Spain), FPH and FO was formulated at a ratio of (1:2:2:2 *w*/*w*/*w*/*w*). The carrageenan, egg white and FPH were previously dissolved in warm water (ratio 1:10 *w*/*v*), then the FO and NaCl (0.1% *w*/*w*, used as flavour enhancer) were added and the mixture was homogenised in a T-25 digital Ultra-Turrax (IKA-Werke GmbH& Co., KG, Staufen, Germany) (14,000 rpm, 22 °C, 5 min). The resulting emulsion was immediately spray dried in a Basic Mini Spray-Dryer B290 (Büchi, Flawil, Switzerland) (inlet temperature: 220 °C, aspiration: 90%, pump 65%, flow rate 12.3 L/min); or heat dried in a drying chamber with forced convection (FD 240, Binder, Tuttlingen, Germany) at 45 °C, 60° or 80 °C. The resulting heat-dried cakes were subsequently ground to a fine powder. The spray-dried (SD) and heat-dried (HD-45, HD-60 and HD-80) powders were stored at 4 °C in airtight containers until use.

### 2.5. Properties of Dry Powders

Water solubility (%) and hygroscopicity (%) were determined gravimetrically as previously described [18]. Changes in hygroscopicity were monitored for 30 days at 23 °C and ≈75% relative humidity. The ζ potential (mV) was determined by Electrophoretic Light Scattering (ELS) after dispersing the dry powders in deionised water (1% *w*/*v*) at room temperature, using a Zetasizer Nano (Malvern Instruments, Ltd. Worcestershire, UK). Lipid oxidation was determined by the spectrophotometric method of thiobarbituric acid-reactive substances (TBARS) measurement, according to Gómez-Estaca et al. [23], using a standard curve prepared with 1,1,3,3-tetraethoxypropane. Results were expressed as mg of malonyldialdehyde (MDA)/kg of dry powder. The dry powders were prepared in triplicate, and all determinations were carried out at least in triplicate.

### 2.6. Preparation of the Ready-to-Eat (RTE) Product

Two kg of each of the three types of fish mince were semi-thawed and mixed in a mixer machine (Mainca, Barcelona, Spain) in a ratio of 1:1:1 (*w*/*w*/*w*) (step S1). Half of the blend was homogenised with 0.5% NaCl (*w*/*w*) and crushed ice for 2 min in a Stephan UM12 (Hameln, Germany) under vacuum and refrigeration to obtain a dough (salt-ground muscle). The dry powder (SD or HD-80) was then added to the dough at a concentration of 8% (*w*/*w*) and homogenised for 30 s. The intended final moisture content in the homogenate was set at 76%. The dough was incorporated into the reserved half of the fish mince blend already thawed, and both were mixed for 3 min in a mixer machine (Mainca, Barcelona, Spain) (step S2). The resulting batter was moulded into portions of about 80 g (7.5 cm × 7.5 cm × 1 cm), which were subjected to a mild heat treatment at 60 °C for 40 min in the drying chamber (FD 240, Binder, Tuttlingen, Germany) (step S3). In order to provide the final ready-to-eat condition, the resulting product was then placed on a griddle at high heat (≈90 °C) for 1 min on each side (step S4), using a few drops of sunflower oil. Three replications of the experiment have been carried out. The product was stored at 4 °C until use for analysis (within 1–2 days).

### 2.7. Shear Strength and Water Holding Capacity (WHC)

Shear strength was determined using two rectangular moulded pieces per sample, which were spread on a Kramer cell perpendicular to the cell grooves, using a TA-XT2 Texture Analyser (Texture Technologies Corp., Scarsdale, NY, USA), with a cell load of 30 kg at 100 mm/min). Shear strength was expressed as N/g of muscle, at the point of maximum load. Results were the average of at least five determinations.

WHC was determined following the centrifugation method described by Gómez-Guillén et al. [24]. WHC (%) was expressed as the amount of retained water per 100 g of water present in the sample before centrifuging. Determinations were carried out at least in triplicate.

### 2.8. Colour Parameters

The colour parameters L* (lightness), a* (redness) and b* (yellowness) were measured using a Konica Minolta CM-3500d colourimeter (Osaka, Japan), equipped with a D65 illuminant and a D10 standard observer. Results were the average of at least 10 replicates.

### 2.9. Lipid Fractionation and Fatty Acid Composition

Fat was extracted from the RTE product using hexane as an organic solvent and subsequently fractionated into neutral lipids, free fatty acids and phospholipids by consecutive elution in chloroform-isopropanol, diethyl ether 2% in acetic acid, and methanol 2% in HCl, respectively, as described in Pascual-Silva et al. [25]. Fatty acid methyl esters (FAME) in each fraction were determined by GC-FID and identified by comparing retention times with those of standards FAME 37 SUPELCO Ref CRM47885 + PUFA no. 2 Animal Source Ref 47015-U Sigma + PUFA no. 3 Menhaden oil Ref 47085-U Sigma [25]. Results were the average of three determinations and were expressed as mg FAME/g of extracted fat.

The polyene index (PI), expressed as the ratio between EPA + DHA and palmitic acid, was calculated for each separate lipid fraction as an indicator of PUFA damage [26].

### 2.10. Microbiological Analyses

Microbiological analyses were carried out in duplicate as previously described by Alemán et al. [27]. Briefly, 10 g of the sample were transferred into sterile bags (Sterilin, Stone, Staffordshire, UK) and mixed with 90 mL of buffered 0.1% peptone water (Oxoid, Basingstoke, UK) in a vertical laminar-flow cabinet (mod. AV 30/70 Telstar, Madrid, Spain). After 1 min shaking in a Stomacher blender (model Colworth 400, Seward, London, UK), appropriate dilutions were prepared for the following determinations: total bacterial counts (TBC) on pour plates of Plate Count Agar (PCA) incubated at 30 °C/72 h; *Enterobacteriaceae* on double-layered plates of Violet Red Bile Glucose agar (VRBG, Oxoid) incubated at 30 °C/48 h; lactic acid bacteria on double-layered plates of De Man, Rogosa and Sharpe (MRS) agar (Oxoid) incubated at 30 °C/72 h; *Staphylococcus* coagulase positive (*S. aureus* and other species) on Baird Parker-RPF agar supplemented with rabbit plasma and fibrinogen (BioMérieux S.A., Marcy l’Etoile, France). Microbiological counts were expressed as the log of the colony-forming units per gram (Log cfu/g) of sample.

### 2.11. Sensory Analysis

Twelve previously trained panellists were chosen for this study. The attributes selected in the final RTE product, on a scale of 0 to 5, were: juiciness (0 very dry; 2.5 mild; 5 excessively), off-flavours (fish protein hydrolysate and/or fish oil) (0 absence; 2.5 mild; 5 strong), fish-flavour (0 absence; 2.5 mild; 5 strong), texture (0: soft; 2.5 mild; 5: hard), overall acceptability (0 not acceptable, 5 very acceptable).

### 2.12. Statistical Analyses

The experiments, both for objectives (i) and (ii), were repeated three times independently. Each analytical determination was carried out using different replicates, as described in the corresponding sections in M&M. Results are the averages and standard deviations for the three independent repeated experiments. Analysis of variance using the average values obtained for each of the three independent repeated experiments was performed using the SPSS^®^ computer program (IBM SPSS Statistics 26 Software, Inc., Chicago, IL, USA). Differences between means were assessed by the Duncan test, with a significance level set at *p* ≤ 0.05.

## 3. Results and Discussion

### 3.1. Properties of Dry Powders

Spray drying (SD) and heat drying (HD) methods were compared in order to obtain a dry powdered ingredient based on the mixture of fish oil entrapped in a matrix of κ-carrageenan, egg white and a fish protein hydrolysate. In the case of the HD powders, different drying temperatures (45 °C, 60 °C and 80 °C) were also studied in order to evaluate their influence on water solubility, surface charge of the rehydrated colloidal dispersion (ζ potential) and lipid oxidation (TBARS) (Table 1).

The water solubility of the HD powders decreased significantly (*p* ≤ 0.05) with increasing drying temperature, which could be largely attributed to the decrease in protein solubility due to the progressive thermal aggregation of egg white proteins, as well as to the heat-induced protein-carrageenan interactions [14,15]. The water solubility of HD80 was considerably lower than that of SD, the latter showing values closer to those of sample HD60. Similar findings were previously reported for comparable SD and HD powders prepared with salt-soluble fish muscle protein instead of egg white, suggesting a higher prevalence of soluble complexes in the spray-dried powder [18]. Furthermore, the water solubility of HD80 with egg white was even lower than that reported for muscle protein in the referred work (34.68 vs. 46.69%, respectively). The low solubility of HD80 would, in principle, be adequate to prevent undesirable flavours associated with an early release of fish oil during food processing or storage [16]. After rehydration, all powders studied led to stable colloidal dispersions with high electronegative ζ potential, which showed a slight tendency to increase (*p* ≤ 0.05) (net values) with increasing drying temperature in the HD samples, and with no significant differences (*p* > 0.05) between HD60 and SD. These results indicate a strong inverse relationship between surface charge and water solubility, in line with the progressive heat-induced protein and protein-carrageenan aggregation. According to McClements [28], ζ potential values greater than ± 30 mV promote electrostatic repulsion among microparticles, indicating high colloidal stability. The ζ potentials in the present SD and HD80 dispersions with egg white were more electronegative than those of the corresponding fish muscle protein samples previously reported [18]. The conversion of fish oil into a stable colloidal form without oil phase separation in all samples suggests a successful entrapment within the protein-carrageenan matrix.

The extent of lipid oxidation in the dry powders was evaluated in terms of TBARS formation (Table 1). The SD powder presented considerably greater (*p* ≤ 0.05) TBARS than any of the HD powders, attributed to the higher exposure to oxygen during atomisation. Lipid oxidation in microencapsulated fish oil has been reported to occur both during emulsion formation and during the spray drying process [29]. The increasing drying temperature in the HD samples led to a decrease (*p* ≤ 0.05) in TBARS, which was more pronounced between HD45 and HD60. The decrease in lipid oxidation in the HD samples as a function of increased drying temperature had already been reported in a study using fish muscle protein instead of egg white [18] and was attributed to the progressive formation of a protein gel matrix during water evaporation, which prevented fish oil from being exposed to oxygen.

**Figure 1:**
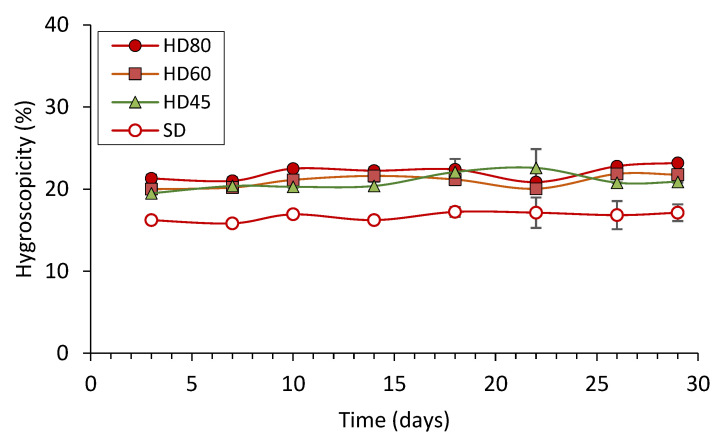
Hygroscopicity (%) of spray-dried (SD) and heat-dried powders obtained at 45 °C (HD45), 60 °C (HD60) and 80 °C (HD80). The hygroscopicity was determined for 30 days at 23 °C and ≈75% relative humidity. Average ± standard deviation; three independent experiments (*n* = 3).

The hygroscopicity of the dry powders was measured during 30 days of storage at a relative humidity of about 75% (Figure 1). The SD powder showed lower (*p* ≤ 0.05) hygroscopicity than the HD powders throughout the whole storage period, with no evident differences based on the drying temperature of the latter. Compared with the aforementioned work carried out with fish muscle protein [18], the present SD and HD powders made with egg white (irrespective of the drying temperature) were much less hygroscopic and more stable during storage. The main difference in water absorption with respect to the reported work lies in the greater hydrophilic condition of the salt-soluble fish muscle protein with residual NaCl, in contrast to the highly soluble dehydrated egg white used.

Even so, hygroscopicity values in the SD and HD powders in the present study were approximately two-fold higher than those reported for other protein/polysaccharide mixtures encapsulating fish oil [30,31]. This difference could be attributed to the contribution of the fish protein hydrolysate, in terms of its high surface-active properties and relatively high ash content [18], in addition to the 0.1% added salt used as a flavour enhancer in the present work. Based on the lower water solubility and lipid oxidation, HD80 powder could be the most interesting candidate for incorporation into the formulation of the restructured fish product. For comparison purposes, however, the effects of adding the SD powder were also evaluated.

### 3.2. Properties of Fish Mince

The proximate composition of the three types of fish mince shown in Table 2 depicts the great variations in fat content, which ranged from 11.5% in HM to 0.9% in P, with GG showing intermediate values (6.2%). The moisture content was inversely related to the fat content in the different species. Protein amounts differ slightly (*p* ≤ 0.05) within the three types of fish mince, in contrast, ash content showed no significant differences.

**Figure 2:**
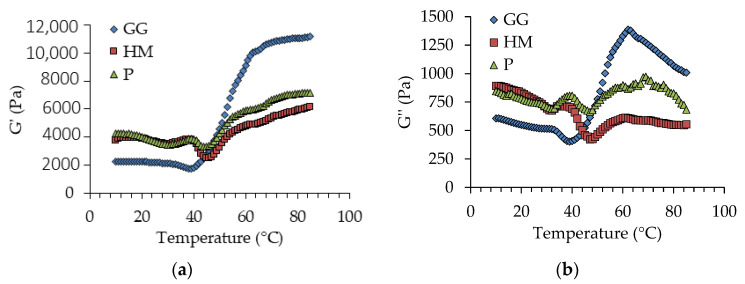
Changes in (**a**) elastic modulus G′, and (**b**) viscous modulus G″, of salt-ground batters from the different fish mince (GG: grey gurnard, HM: horse mackerel; P: pouting) as a function of heating temperature at 1 °C/min. Average values; three independent experiments (*n* = 3).

Differences in the thermal protein aggregation capacity of the studied fish species were assessed by changes in the viscoelastic properties (elastic modulus G′; viscous modulus G″) of the corresponding salt-ground muscles as the heating temperature increased from 10 °C to 85 °C (Figure 2).

The thermal profile of GG showed much lower G′ and G″ values between 10 °C and 40 °C than those of P and HM, indicating a higher degree of salt-induced protein unfolding in GG, which would favour the subsequent formation of intermolecular bonds. In fact, the sharp increase in G′ and G″ from 38 °C to about 60 °C in this fish mince denoted a pronounced rise in protein aggregation. By increasing the heating temperature, the further moderate increase in G′, in contrast to the decrease in G″ values, indicated that the gel was mostly formed at about 60 °C and that only a slight reinforcement took place at higher temperatures [32]. The thermal profiles of P and HM were characterised by presenting relative G′ and G″ peaks at around 37 °C, followed by a decrease in the viscoelastic parameters reaching a minimum at 46 °C. These events corresponded to the so-called “setting” and “*modori*” phenomena described for some fish species, associated, respectively, with the preliminary formation of an ordered protein network stabilised by relatively strong protein–protein interactions and followed by the destabilisation of the network strongly induced by the activity of heat-stable proteases [33]. It should be noted that a considerable amount of sarcoplasmic proteins (including enzymes) could be present in the fish mince, as no washing step was carried out during the preparation of the mince in order to preserve the original fat content and avoid residual protein generation. The lower G′ and G″ values of P and HM at 60 °C and higher temperatures, compared with those of GG, indicate their lower protein-gelling capacity. In the case of horse mackerel, both the high fat content and the more intense *modori* associated with the higher amount of dark muscle could have greatly contributed to interfering with protein aggregation [34]. In contrast, the relatively low gelling capacity of P, which was leaner compared with GG, could most likely be ascribed to the more aggregated initial state of the salt-ground muscle protein. In view of the results of the thermal profile of protein gelation, 60 °C was selected as the heating temperature to produce the pre-formed fish portion in order to avoid overheating and nutrient loss, especially with regard to the fish oil.

### 3.3. Preparation of the RTE Product

**Figure 3:**
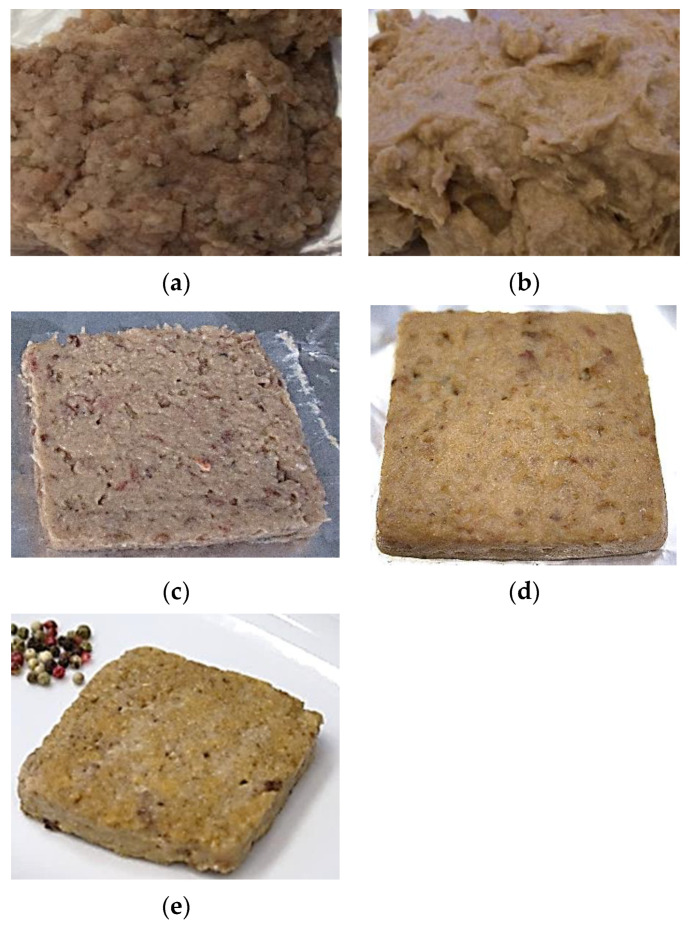
Changes in visual appearance during the successive processing steps: (**a**) raw fish mince mixture (S1); (**b**) salt-ground muscle with dry powder; (**c**) moulded blend of raw mince and salt-ground batter containing the dry powder (S2); (**d**) pre-heated portion at 60 °C (S3); (**e**) final grilled portion (RTE product, S4).

The restructured fish product was based on the mixture of three equal parts of mince of the fish species studied, based mainly on the high gelling capacity and intermediate fat content of GG, and the higher fat content of HM. First, the semi-thawed minced muscles of the three species were mixed to obtain a homogeneous coarse fish mince (Figure 3a).

Half of the mixture was homogenised with NaCl to obtain a fine salt-ground muscle, to which the corresponding dry powder was subsequently added and homogenised (Figure 3b). The low solubility of powders SD and HD80, especially of the latter, would be desirable in this step to avoid oil release from microparticle disintegration or solubilisation when in contact with the wet salt-ground muscle during mixing. An amount of 8% dry powder in the batter was selected as the adequate amount so as not to impair the flavour or juiciness of the final product. The dough formed by salt-denatured muscle protein containing the dry ingredient was then carefully blended with the reserved mixture of raw mince, resulting in a homogeneous and easy-to-mould blend (Figure 3c), in which the salt-denatured protein acted as a binding agent of minced fish during moulding and upon ulterior heating. This arrangement reduced the NaCl content required for adequate myofibrillar protein solubilisation prior to thermal gelation, which was carried out by heating at 60 °C (Figure 3d), resulting in a final portion of slightly grilled fish with a more natural palatability and appearance (Figure 3e).

The process to obtain the RTE product was therefore divided into four main steps (S1–S4), during which, changes in shear strength, water-holding capacity, and colour parameters (CIE Lab *) were monitored (Table 3).

As expected, the moulded blend (S2) (raw fish mince + salt-ground muscle + powder HD80 or SD) showed a marked decrease in shear strength with respect to the raw fish mince (S1), attributed to the solubilisation of myofibrillar proteins in the salt-ground muscle. The type of dry powder did not induce any significant difference (*p* ≤ 0.05) in this step. Likewise, pre-heating the moulded blend to 60 °C increased the strength of the intermediate product (S3) in a similar way in HD80 and SD, mainly due to muscle protein aggregation in the raw fish mince and gelation in the salt-ground muscle. The greatest increase in product firmness occurred after the final step (S4), which completed the cooking treatment and provided the ready-to-eat-property to the final product (see Figure 3e), with no significant differences (*p* > 0.05) depending on the dry powder added.

The raw mince mixture (S1) presented a relatively high water-holding capacity (WHC) (around 72%), as compared to values reported in frozen fish mince from haddock or Atlantic mackerel [10], or from horse mackerel (*Trachurus trachurus*) [35]. This finding indicates that the unwashed mince mixture retained good protein functionality, taking into consideration the previous manipulation carried out to obtain the respective types of fish mince, freezing without cryoprotectants, partial thawing and chopping to obtain the mince mixture. WHC values increased considerably in the subsequent step, S2, which is attributed to the water entrapped by the salt-ground muscle and the partial hydration of the carrageenan–protein complex in the dry powders added during this step. This evidences the dual function of the complex, i.e., as a fish oil entrapping agent and as a water binder. The increase in WHC was much more pronounced (*p* ≤ 0.05) in SD compared with HD80, which could be related to the higher water solubility of the former, making both κ-carrageenan and egg white protein more available to trap surrounding water molecules. Despite this pronounced difference in the S2 blend based on the type of powder used, the WHC of the respective intermediate heated S3 products was not significantly different (*p* > 0.05). In this step, the partial protein gel network induced upon heating at 60 °C was mainly responsible for the entrapment of water molecules and was practically unaffected by the type of dry powder incorporated. In the final RTE products (S4-HD80 and S4-SD), the WHC slightly decreased (*p* ≤ 0.05) with respect to their S3 counterparts, probably due to partial surface dehydration and protein aggregation resulting from the grilling step. In this respect, the final moisture content after grilling was 65.14 ± 0.84% in S4-SD and 69.00 ± 0.27% in S4-HD80. In spite of this, the similar (*p* > 0.05) WHC value of ≈ 80% in both RTE products indicates that water was properly entrapped with no signs of exudation.

The product also exhibited obvious colour changes during the different processing steps (Table 3). The lightness (L*) values increased notably from S1 to S2 with both added powders, which is attributed to the slight whitening that the fish muscle protein homogenate exhibits when solubilised with NaCl (see Figure 3b). The heat-induced protein aggregation in S3 and S4 caused a minimal increase (*p* ≤ 0.05) in L* values with respect to S2 in the samples containing HD80. In contrast, the addition of SD decreased product luminosity (*p* ≤ 0.05) upon heating, leading in fact to lower L* values with respect to their HD counterparts. The low positive a* values generally indicated a slight tendency to redness, with large relative variations attributed to the intrinsic heterogeneity of the samples. The significant increase (*p* ≤ 0.05) in redness found in the RTE product with the SD powder after the grilling step is noteworthy, as it was not as evident in the product with HD80. The grilling effect in both S4-HD80 and S4-SD was also significant (*p* ≤ 0.05) in terms of yellowness (b*). The noticeable changes in colour parameters that occurred in S4 with both types of dry powders were the result of partial desiccation and browning on the surface of the fish portion upon grilling. The slight differences found between SD- and HD80-added samples could be mainly caused by the different water solubility of the respective dry powders, which probably influences to some extent their hydration and mixing with the muscle protein, as well as the heat-induced dehydration in the last processing step.

### 3.4. Microbiological Analyses

In general, the type of dry powder incorporated did not induce major differences in the final product and throughout the various preparation steps, with the exception of S2-SD, which significantly increased the WHC, leading to certain colour changes, probably related to greater rehydration or solubilisation in contact with the wet muscle dough. Therefore, the formulation that included the SD powder was selected to characterise the changes in microbial growth during the different steps of fish product preparation (Table 4). Initially, the microbial load in the S1 step was 4.6 Log cfu/g, which is within the regular range of counts obtained in a fresh market fish subjected to handling [36]. *Pseudomonads* constituted the largest group of the total flora, as they may be one of the predominant groups present in chilled fish due to their psychrotrophic character. *Enterobacteriaceae* were below the recommended detection limit for fishery products (Decision 95/149/EC, DOCE L.97 29/4/95), while lactic acid bacteria were also present, with counts of 2.7 log CFU/g (Table 4).

S2 recorded total bacteria counts similar to those of the starting material (minced muscle) (*p* > 0.05), indicating good practices during the processing steps where the mixing of ingredients and homogenisation are necessary to obtain the product. Thus, the slight increase in WHC observed at the S2 step in this preparation did not increase the bacteria counts with respect to the initial load. The heating step at 60 °C (S3) induced a small decrease in most of the studied groups (≤1 log cycle, *p* ≤ 0.05). The time/temperature selected for this process step, similar to pasteurisation, was not enough to successfully eliminate microorganisms. This fact could be due to the near-neutral pH of the product obtained and the conventional (non-sterile) handling of the product when it was removed from the oven. However, the decrease was notable after grilling (S4), dropping by about two logarithmic units for most of the microbial groups, which suggests that this heat treatment was particularly effective in reducing the bacterial load. The aerobic mesophilic bacteria count in the S4-SD final product was 2.19 ± 0.10 log CFU/g, very close to that determined for comparison purposes in the final product S4-HD80 (1.82 ± 0.24 log CFU/g), indicating again the close resemblance between both types of product subjected to the same culinary treatment. It is considered that the microorganisms developed mainly from inside the product (where heat did not reach as easily and thus was not sufficient to reduce the microbial load) and, as in the previous case (S3), due to the standard cooking and plating conditions in which the environment was non-sterile. The coagulase-positive staphylococci counts remained below detection limits in the different treatments, which implies the absence of cross-contamination during the fillet processing as well as during subsequent processes.

### 3.5. Compositional Properties of the RTE Product

Based on the few differences observed during the product development, and the slightly lower microbial load in the final product, S4-HD80 was selected for further analyses in terms of proximate composition and fatty acids. The total protein, fat, moisture and ash contents were, respectively, 18.50 ± 0.04%, 10.40 ± 0.02%, 69.00 ± 0.27% and 1.5 ± 0.27%. The theoretical carbohydrate content could be estimated to be around 0.5%, corresponding to the amount of κ-carrageenan added as a wall material for fish oil microencapsulation.

Table 5 presents the results of the fractionation of the total fat extracted into three main classes based on their different polarity, i.e., neutral lipids (NL), free fatty acids (FFA) and phospholipids (PL), as well as the fatty acid composition of each fraction studied. Based on the fatty acid content of each separated lipid fraction from the final RTE product, the amount of NL was clearly predominant, with 91% of the total fatty acids, while FFA and PL accounted for 5% and 4%, respectively.

Concerning all lipid fractions, the most abundant fatty acids were oleic acid (C18:1n9c), palmitic acid (C16:0) and DHA (C22:6n3). Additionally of note were the amounts, in descending order, of palmitoleic acid (C16:1n7), linoleic acid (C18:2n6c) and EPA (C20:5n3) in the NL fraction. The RTE product was rich in mono- and polyunsaturated fatty acids, which represented 75% of the total FA in the predominant NL fraction. The sum of these FA accounted for 65% of the FFA fraction, compared to 48% in the PL fraction. Considering the sum of the fractions (NL + FFA + PL), unsaturated fatty acids represented 73.5% of the total fat extracted. In particular, the significant amount of long-chain ω-3 fatty acids EPA and DHA (15.5% of total fatty acids) that are part of all lipid fractions is known to promote positive effects on cardiovascular health [37]; this assumption would depend on the consumer’s daily intake.

The polyene index (PI) was 0.84 in neutral lipids, 1.57 in FFA and 1.72 in phospholipids, respectively. This lower value found in the most abundant NL fraction extracted from the RTE product was in line with the higher proportion of unsaturated fatty acids, which are prone to oxidative changes. The PI calculated for the sum of NL + FFA + PL was found to be 0.94. This value was considerably lower than that found in lipids extracted from raw horse mackerel patties [38], suggesting that lipid degradation could have taken place possibly during the processing/cooking of the restructured fish product. Similarly, the n3/n6 ratio in the predominant neutral fraction (2.18) and in the sum of fractions (2.53) may have been adversely affected by heating, in agreement with Bhouri et al. [39]. Nevertheless, n3/n6 ratios between two and five have been shown to exert suppressive effects on cancer, and cardiovascular and inflammatory diseases [40], depending on daily intake. So, in principle, the restructured product can be considered healthy in terms of fat composition, and also in protein content, which is relatively high, similar to the typical composition of fish muscle.

A sensory analysis was carried out to obtain basic information on the general acceptability of the ready-to-eat product and, more specifically, on its juiciness, texture, fishy flavour and presence of off-flavours (fish protein hydrolysate and/or fish oil). For this purpose, the product was preheated before serving. On a scale of 0 to 5, both juiciness and texture recorded medium scores (2.4 ± 0.4 and 3.5 ± 0.3, respectively), which could be associated with a positive ideal perception. The almost absence of off-flavours was observed (0.9 ± 0.1), and a fishy taste was evident (4.1 ± 0.2). It should be noted that the overall acceptability scored quite high (4.2 ± 0.27), which may lead to a real option as a potentially functional commercial product.

## 4. Conclusions

The mixture of egg white protein, κ-carrageenan and a fish protein hydrolysate was effective for fish oil entrapment, which, after spray drying or heat drying in an oven, resulted in a powder of low water solubility, especially when using the heat drying method at a high temperature (80 °C). Contrary to what might be expected, increasing the drying temperature from 45 °C to 80 °C considerably decreased the degree of oxidation of fish oil, due to the protective effect of the matrix formed during the aggregation of egg white protein and carrageenan. In contrast, the spray-dried powder showed greater lipid oxidation and lower hygroscopicity.

Despite the differences between powders SD and HD80, very slight deviations were observed in the respective restructured fish products throughout the successive processing steps. Furthermore, the mixture of fish mince from species with very different fat contents and gel-forming capacity proved to be a suitable basis for the development of the restructured product, using half of the mix in the form of a salt-ground batter to favour the overall binding property among minced particles upon heating. The presence of k-carrageenan and egg white protein also led to maintaining the WHC and preventing syneresis. The final short grilling step, in addition to providing increased firmness to the ready-to-eat product, contributed greatly to decreasing the microbial load, which could lead to a longer shelf life. The final product could be considered a functional food based on its high protein content, in line with typical values found in raw fish muscle, as well as on its high amount of polyunsaturated fatty acids, including the well-regarded long chain ω-3 fatty acids.

## Figures and Tables

**Table 1:** Properties of spray-dried (SD) and heat-dried powders obtained at 45 °C (HD45), 60 °C (HD60) and 80 °C (HD80).

	Water Solubility (%)	ζ Potential (mV)	TBARS mg MDA/kg
HD45	73.01 ± 0.88 a	−41.96 ± 6.67 a	160.3 ± 1.03 a
HD60	52.43 ± 0.35 b	−50.14 ± 2.83 b	81.14 ± 1.37 b
HD80	34.68 ± 0.58 c	−57.60 ± 3.24 c	64.18 ± 2.79 c
SD	58.25 ± 3.37 d	−51.04 ± 0.97 b	242.5 ± 1.99 d

Different letters (a,b,c,…) in the same column indicate significant differences (*p* ≤ 0.05). Average ± standard deviation; three independent experiments (*n* = 3).

**Table 2:** Proximate composition of individual fish minces from pouting fish (P), grey gurnard (GG) and horse mackerel (HM).

	Moisture (%)	Ash (%)	Fat (%)	Protein (%)
P	78.4 ± 0.1 a	1.55 ± 0.1 a	0.9 ± 0.04 a	19.13 ± 0.73 a
GG	72.3 ± 0.6 b	1.01 ± 0.58 a	6.2 ± 0.05 b	21.74 ± 0.23 b
HM	66.7 ± 1.8 c	1.16 ± 0.03 a	11.5 ± 0.07 c	20.64 ± 0.51 c

Different letters (a,b,c,…) in the same column indicate significant differences (*p* ≤ 0.05). Average values ± standard deviation; three independent experiments (*n* = 3).

**Table 3:** Changes in shear strength, water-holding capacity (WHC) and colour parameters during the successive processing steps.

	Shear Strength (N/g)	WHC (%)	L*	a*	b*
S1	12.51 ± 1.85 a	71.87 ± 0.23 a	43.97 ± 3.36 a	1.16 ± 0.47 a	12.37 ± 1.72 a
S2-HD80	3.61 ± 0.28 b	78.03 ± 0.48 b	50.71 ± 1.17 b	2.92 ± 0.24 b	16.46 ± 1.12 bc
S3-HD80	6.07 ± 0.65 c	81.92 ± 0.11 cd	52.79 ± 0.67 c	1.85 ± 0.23 c	16.69 ± 0.86 c
S4-HD80	21.82 ± 1.29 d	80.22 ± 0.96 e	53.17 ± 2.11 c	3.05 ± 0.30 b	20.76 ± 0.30 d
S2-SD	3.76 ± 0.67 b	95.10 ± 0.25 f	50.91 ± 1.22 b	2.79 ± 0.24 b	15.66 ± 0.61 b
S3-SD	5.46 ± 0.88 c	82.42 ± 0.13 d	46.50 ± 1.33 d	2.86 ± 0.26 b	16.05 ± 0.74 bc
S4-SD	20.48 ± 1.18 d	80.95 ± 0.33 ce	48.24 ± 2.06 e	4.94 ± 1.51 d	19.72 ± 1.93 e

S1: Raw mince mixture; S2: moulded blend of raw mince plus salt-ground batter with dry powder; S3: pre-heated portion at 60 °C; S4: grilled portion (RTE); -SD: with addition of spray-dried powder; -HD80: with addition of heat-dried powder at 80 °C. Different letters (a,b,c,…) in the same column indicate significant differences (*p* ≤ 0.05). Average ± standard deviation; three independent experiments (*n* = 3).

**Table 4:** Microbiological analyses (Log CFU/g) during the successive processing steps including the spray-dried powder (SD).

Sample	Total Bacterial Count	*Enterobacteriaceae*	Lactic Acid Bacteria	*Pseudomonads* spp.	*Staphylococcus* Coagulase +
S1	4.64 ± 0.14 a	2.9 ± 0.06 a	2.73 ± 0.02 a	3.86 ± 0.08 a	<2
S2-SD	4.72 ± 0.12 a	3.05 ± 0.06 a	2.51 ± 0.11 b	4.12 ± 0.02 b	<2
S3-SD	3.84 ± 0.62 b	2.34 ± 0.05 b	2.37 ± 0.06 c	3.82 ± 0.22 a	<2
S4-SD	2.19 ± 0.10 c	1.00 ± 0.00 c	1.60 ± 0.00 d	2.00 ± 0.00 c	<

S1: Raw mince mixture; S2: moulded blend of raw mince plus salt-ground batter with spray-dried powder; S3: pre-heated portion at 60 °C; S4: grilled portion (RTE). Different letters (a,b,c,…) in the same column indicate significant differences (*p* ≤ 0.05). Average ± standard deviation; three independent experiments (*n* = 3).

**Table 5:** Fatty acid composition of fat extracted from the finished RTE product (S4-HD80) and subsequently fractionated into neutral lipids (NL), free fatty acids (FFA) and phospholipids (PL).

Fatty Acids	NL (mg/g Fat)	Free Fatty Acids (mg/g Fat)	Phospholipids (mg/g Fat)
C14:0	13.23 ± 0.06	0.36 ± 0.03	-
C14:1n5	0.98 ± 0.01	-	-
C15:0	1.47 ± 0.01	-	-
C16:0	70.06 ± 0.35	4.93 ± 0.15	4.54 ± 0.05
C16:1n7	29.21 ± 0.15	0.76 ± 0.04	0.34 ± 0.02
C16:2n4	1.32 ± 0.08	-	-
C17:0	2.07 ± 0.00	-	-
C18:0	19.16 ± 0.12	2.46 ± 0.16	2.49 ± 0.05
C18:1n7c	15.04 ± 0.10	0.71 ± 0.03	0.62 ± 0.00
18:1n9c	173.01 ± 1.09	3.95 ± 0.70	2.80 ± 0.05
C18:2n6c	28.57 ± 0.16	0.63 ± 0.10	0.39 ± 0.02
C18:3n3	2.12 ± 0.15	-	-
C18:4n3	2.36 ± 0.02	-	-
C20:0	1.11 ± 0.01	-	-
C20:1n9	4.41 ± 0.05	-	-
C20:2n6	0.70 ± 0.00	-	-
C20:3n3	0.72 ± 0.07	-	-
C20:4n3	1.77 ± 0.00	-	-
C20:4n6	3.26 ± 0.02	0.56 ± 0.18	0.43 ± 0.00
C20:5n3	23.91 ± 0.16	2.40 ± 0.15	1.02 ± 0.00
C22:0	1.50 ± 0.01	-	-
C22:1n9	0.79 ± 0.02	-	-
C22:4n6	0.57 ± 0.04	-	-
C22:5n3	6.20 ± 0.02	0.53 ± 0.08	0.53 ± 0.03
C22:6n3	35.17 ± 0.28	5.33 ± 0.26	6.77 ± 0.10
C24:0	0.64 ± 0.03		
TOTAL	439.34 ± 2.50 91%	22.62 ± 1.06 5%	19.93 ± 0.19 4%
∑PUFA	106.67 ± 0.61	9.45 ± 0.05	9.14 ± 0.15
∑MUFA	223.43 ± 1.38	5.42 ± 0.77	0.36 ± 0.07
∑SFA	109.23 ± 0.51	7.75 ± 0.34	7.03 ± 0.05
EPA + DHA	59.08	7.73	7.80
n3/n6	2.18	6.91	0.82
PI	0.84	1.57	1.72

PI: polyene index. Average values ± standard deviation; three independent experiments (*n* = 3).

## Data Availability

Data are contained within the article.
